# Cerebral Venous Thrombosis in Patients With Traumatic Brain Injury: Epidemiology and Outcome

**DOI:** 10.7759/cureus.55775

**Published:** 2024-03-08

**Authors:** Joao Meira Goncalves, Vasco Carvalho, António Cerejo, Patricia Polónia, Elisabete Monteiro

**Affiliations:** 1 Neurosurgery Department, Centro Hospitalar Universitário de São João, Porto, PRT; 2 Faculty of Medicine, Oporto University, Oporto, PRT; 3 Intensive Care Medicine Department, Centro Hospitalar Universitário de São João, Porto, PRT

**Keywords:** secondary intracranial hypertension, cerebral venous infarction, therapeutic anticoagulation, traumatic brain injury(tbi), cerebral venous thrombosis (cvt)

## Abstract

The natural history and epidemiological aspects of traumatic cerebral venous thrombosis (CVT) are not fully understood. Due to the concomitant occurrence with intracranial hemorrhages, guidelines for medical treatment have been highly controversial. In this study, our objective was to carry out an analysis description of the population and to conduct a literature review. A prospectively gathered radiology registry data of patients hospitalized at the tertiary hospital of Centro Hospitalar Universitário do São João, Porto, Portugal, between 2016 and 2021 was carried out. All patients with traumatic brain injury (TBI) and concomitant CVT were identified. CVT was confirmed by CT venogram. Demographic, clinical, and radiological data and their medical management were reported. In-hospital complications and treatment outcomes were compared between patients measured by the Glasgow Outcome Score Extended (GOSE) at discharge and GOSE at three months. There were 41 patients with traumatic CVT admitted to this study. The majority (45.2%) had a hyperdense signal near the lateral sinus at admission, and only 26.2% presented with skull fractures. Of this cohort, 95% had experienced lateral sinus thrombosis. Twenty-five patients (60%) had occlusive venous thrombosis. Venous infarct was the main complication following CVT. Thirty-two patients (78%) were anticoagulated after CVT and four developed complications. At the three-month follow-up after discharge, 28.2% had good recovery (GOSE > 6). This study revealed a higher incidence of CVT in severe TBI and a mild association with skull fractures. There is a higher incidence of CVT in the lateral sinus. Management was inconsistent, with no difference in outcome without or with anticoagulation. Larger, prospective cohort studies are required to better comprehend this condition and determine evidence-based guidelines.

## Introduction

The development of cerebral venous sinus thrombosis (CVST), resulting from a closed head injury (CHI) - with or without skull fractures or intracranial hematoma - has been increasingly reported in recent literature. It is frequently observed within 24 hours of trauma but can be delayed for several days or even weeks [1,2,3]. The pathogenesis of CVST associated with traumatic brain injury (TBI) is not well understood. Numerous hypotheses have been proposed, including skull fractures or intracranial hematoma causing thrombosis through direct compression of the sinus, endothelial damage within the venous sinus leading to activation of the coagulation cascade, intramural hemorrhage due to rupture of small sinusoids, extension of a thrombus from veins of injured emissaries, and compression of the sinuses due to intracranial edema [3]. The prevalence following head trauma is uncertain, but most cases of acute post-traumatic CVST are associated with a skull fracture occurring near a dural sinus. Conversely, late sinus thrombosis can also occur without a skull fracture [3]. Because CVST presents with a diverse clinical spectrum and can be asymptomatic, it may go unrecognized during the index hospitalization until later complications warrant medical attention [4].

Imaging plays an increasingly significant role in the diagnosis and treatment of CVST. A plain head computed tomography (CT) without contrast can detect an intravenous thrombus (delta sign) or lead to an indirect suspicion of it with ischemic or vascular changes due to cerebral venous outflow blockade. CT venogram (CTV) has a very high sensitivity (95%). Other imaging modalities, such as magnetic resonance imaging (MRI) or cerebral angiogram, can additionally be utilized [5,6,7]. There are some concerns about the routine use of anticoagulants in patients with TBI. Consequently, the role of hyperosmolar therapies (hypertonic saline and mannitol) as the first-line treatment to decrease intracranial pressure has been supported. If the situation continues to deteriorate, systemic anticoagulation, surgical decompression, or endovascular treatments, such as chemical thrombolysis or mechanical thrombectomy, may be considered [5,8]. Even though early diagnosis of this pathology has improved, the management of this condition still poses a significant challenge to the medical community.

## Materials and methods

Study design

This investigation was carried out by employing a retrospective case-control design, utilizing registry data that had been collected in a prospective manner from individuals admitted to the neurosurgical department and intensive care unit (ICU) of Centro Hospitalar Universitário do São João, Porto, Portugal, during the period from January 2016 through December 2021.

Patient selection and data extraction

**Figure 1:**
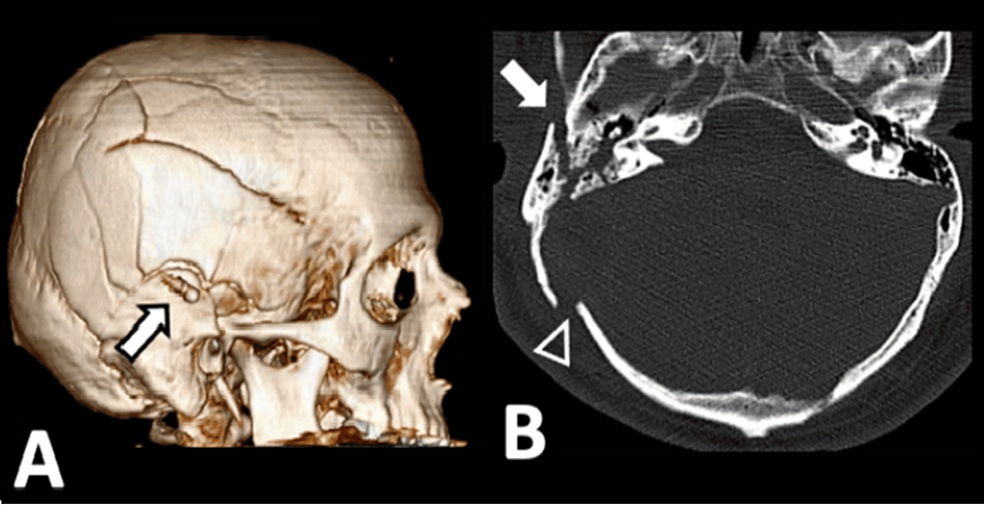
(A) 3D reconstruction of the cranial vault illustrating multiple skull fractures (arrow). (B) CT scan showing bone fractures, concerning the squamous part (arrowhead) and petrous pyramid (arrow) near the right sigmoid sinus.

Eligible participants were identified through a comprehensive review of the hospital’s digital imaging archive system, WebPACS, using the search terms "cerebral venous thrombosis" and "traumatic brain injury." Inclusion criteria were as follows: patients of any age admitted with a diagnosis of TBI, confirmed CVT via CT venogram or angio-MRI, and documented clinical information justifying the imaging request. Exclusion criteria included patients with non-traumatic CVT, incomplete medical records, and those who did not undergo confirmatory imaging studies for CVT (Figure 1).

**Figure 2:**
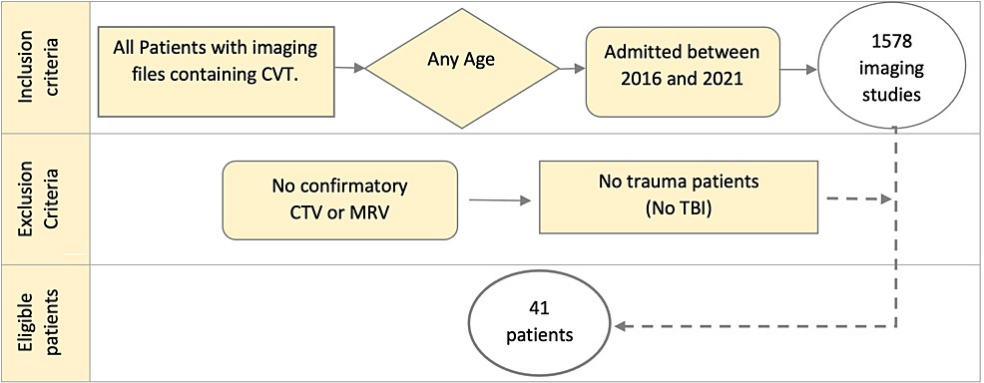
Flowchart demonstrating inclusion criteria. CVT: cerebral venous thrombosis; CTV: CT cerebral venography; MRV: magnetic resonance venography; TBI: traumatic brain injury

A total of 1,578 consecutive imaging studies were initially reviewed. From this pool, patients were excluded based on the absence of TBI in their clinical presentation or lack of confirmatory veno-CT or angio-MRI, leading to the identification of 41 eligible patients for the study cohort (Figure 2).

Data extracted from medical records included demographic information (age and sex), mechanism of injury, use of antithrombotic drugs prior to admission, injury severity scores (Glasgow Coma Scale (GCS) and Marshall scale upon admission), CVT characteristics (location and occlusiveness), additional imaging findings (presence of fractures and infarction), medical and surgical management strategies, and outcomes (including Glasgow Outcome Score Extended (GOSE) at discharge and three months post-discharge). All imaging findings were independently reviewed and reported by a consultant neuroradiologist to ensure accuracy and consistency in interpretation.

Data analysis

Descriptive statistics were employed to summarize the demographic, clinical, and radiological characteristics of the study population. Continuous variables were presented as means and standard deviations or medians and interquartile ranges, depending on the distribution. Categorical variables were summarized as counts and percentages.

The primary outcome measures included the incidence of in-hospital complications and treatment outcomes as measured by GOSE at discharge and GOSE at three months. The study was conducted following ethical guidelines, ensuring the protection of participants' rights and privacy in accordance with ethical standards.

## Results

During the study period, 41 patients were admitted to the neurosurgical department/ICU at Centro Hospitalar Universitário de São João due to head trauma. Among these patients, 28 (68%) were male, and the median age was 50 years. Thirty-eight patients (92.7%) were not taking any medication at the time of admission. Only three patients were taking antithrombotic therapy (either antiplatelet or anticoagulant) upon admission. The most common mechanism of injury was falling from standing height (51.2%) (Table 1).

**Table 1:** Mechanism of injury resulting in venous sinus thrombosis.

Mechanism	Number of patients (n)	Percentage (%)
Fall from standing	21	51.2
Fall from height	13	31.7
Assault (gun or cane)	3	7.3
Road traffic collision (RTC)	2	4.9
Pedestrian versus motorcycle/car	1	2.4
Unknown	1	2.4

Presentation

Upon admission, 25 patients (61%) were classified as having mild TBI (GCS scores between 13 and 15), and only four (9.8%) were classified as having severe TBI (GCS scores between 3 and 8) (Table 2). According to the Marshall scale, 22 patients (53.7%) were categorized as Class 2 (Table 3).

**Table 2:** Glasgow Coma Scale/Score (GCS) at admission Data given in raw number and percent

Admission GCS Score	Number of patients (n)	Percentage (%)
3-8	4	9,8%
9-12	25	61%
13-15	12	29,3%

**Table 3:** Marshall scale/score at admission Data given in raw number and percent

Marshall scale/score	Number of patients (n)	Percentage (%)
Category I	3	7.3
Category II	22	53.7
Category III	5	12.3
Category IV	8	19.5
Category VI	3	7.3

All patients underwent an initial CT scan upon admission. Notably, 35 patients (85%) did not undergo venous-CT within the first 24 hours. Follow-up imaging was performed in 19 patients (46.3%) due to spontaneous hyperdense signal changes near the lateral sinus (transverse and sigmoid). Hypodense signal changes on CT and fractures near the lateral sinus were observed in 11 patients (26.8%) each, respectively. CVTs were most commonly identified in a combination of the sigmoid sinus (SS) and transverse sinus (TS) in 13 patients (31.7%), followed by a combination of SS, TS, and jugular sinus (JS) in approximately 12 patients (29.2%). It was observed that 88% of the patients had at least a sigmoid sinus thrombosis, and 73% had a transverse sinus thrombosis, leading to a lateral sinus thrombosis rate of approximately 95% among all patients admitted to the study.

Management

**Figure 3:**
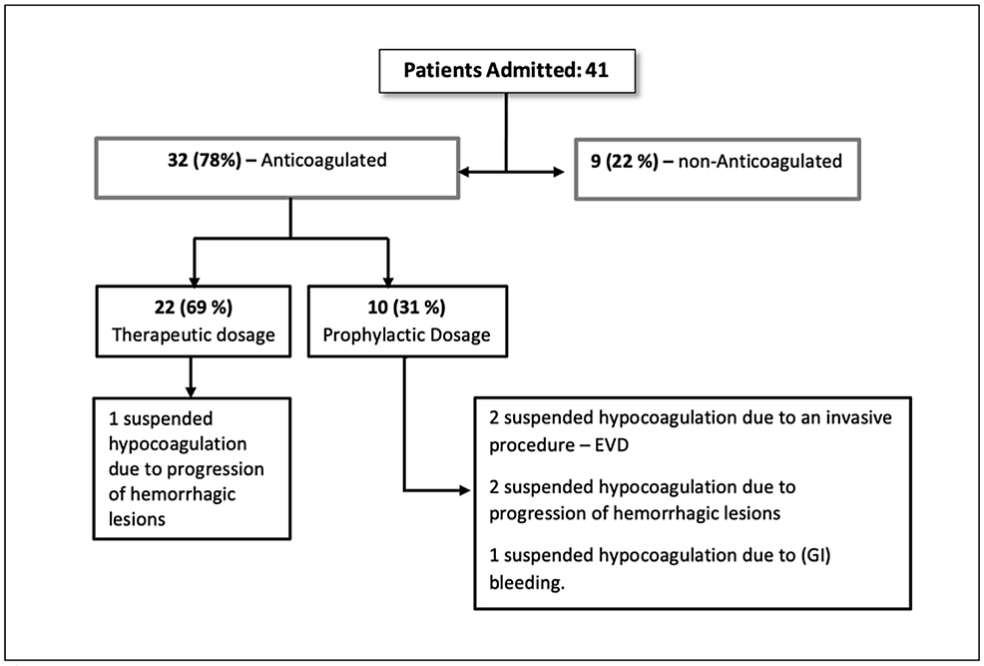
CVT treatment: no patients underwent endovascular treatment. EVD: external ventricular drain; GI: gastrointestinal bleeding

The majority of patients were managed in an ICU setting at some point and were transferred to the neurosurgery ward upon achieving neurological and imaging stability. Only six patients were directly admitted to the neurosurgery ward. Twenty-three patients (56%) were polytraumatized, and the majority sustained blunt-type injuries (95.2%) (Figure 3).

Regarding anticoagulation therapy, nine patients (22%) did not undergo any form of anticoagulation. Ten patients (24%) received prophylaxis with low-molecular-weight heparin (LMWH). Twenty-two patients (53.7%) were initially anticoagulated with therapeutic unfractionated heparin (UFH) and were subsequently treated with LMWH, dabigatran, or warfarin. When therapeutic anticoagulation was deemed necessary, patients were administered UFH without an initial bolus, aiming for activated partial thromboplastin time (aPTT) values between 35 and 40 seconds.

The decision to initiate treatment was determined by the intensive care specialist and consultant neurologist, based on specialist input, clinical judgment informed by consultant-led experience, and the patients’ clinical presentation and imaging findings. Anticoagulation therapy was initiated at a median of five days post-TBI. Complications arising from hypocoagulation were observed within the subsequent two days.

Three patients experienced progression of intracranial hemorrhage, one patient experienced gastrointestinal hemorrhage, and two patients discontinued anticoagulation due to the insertion of external ventricular drains (EVDs). No additional complications were noted.

Morbidity

Of the 41 patients admitted to the study, 25 (61%) experienced occlusive venous thrombosis. Within this subgroup, three patients encountered complications related to the thrombosis: one progressed to vasogenic focal edema, another suffered a venous infarct, and the last one experienced a subsequent intracranial hemorrhage. A total of 16 patients (39%) presented with non-occlusive venous thrombosis, among whom two progressed to venous infarct.

Outcomes

**Figure 4:**
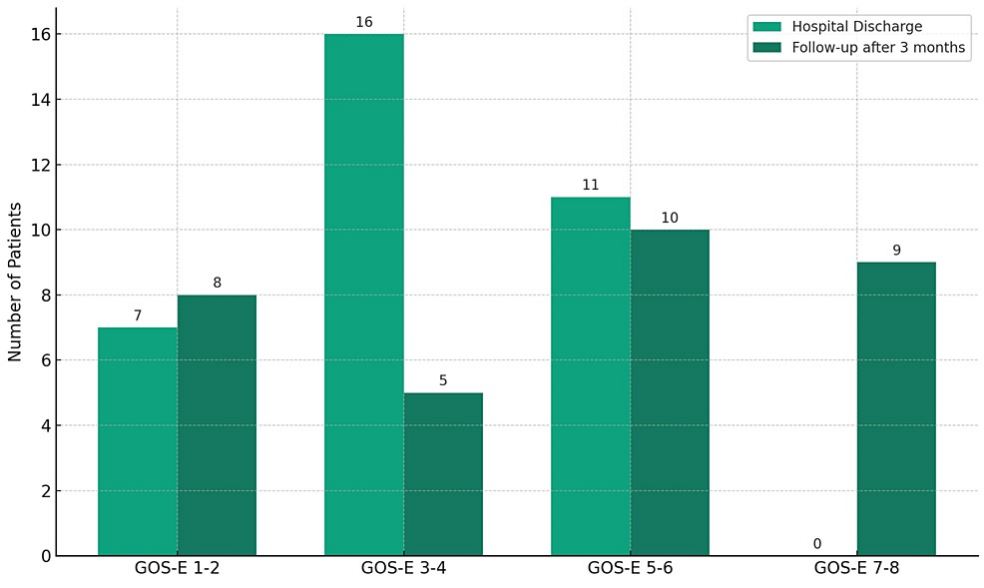
GOS-E at discharge and at last follow-up. GOS-E – Glasgow Outcome Scale Extended

The GCS and GOSE were evaluated at discharge. Five patients succumbed during the study period (four due to progressive neurological deterioration related to the TBI, and one was lost to follow-up). Among the remaining 35 patients, 84.3% had a GCS score ranging from 13 to 15; 3.1% had a GCS score ranging from 9 to 12, and 12.5% had a GCS score ranging from 3 to 8 at discharge (Table 4). The GOSE scores are detailed in (Table 4). Twelve patients (34.3%) exhibited severe disability at discharge. Only six patients (17.1%) had upper moderate disability, and five (14.3%) had lower moderate disability. No patient scored above six points on the GOSE at discharge. However, at the three-month follow-up, an improved functional status was observed: six patients (17.1%) achieved a lower good recovery, and three patients (8.6%) achieved an upper good recovery. Only three patients (8.6%) scored below 3 on the GOSE scale. An additional patient died during the three-month period (Figure 4).

**Table 4:** Outcome at discharge

Characteristic	Number of patients (n)	Percentage (%)
ECG		
3-8	4	11,5
9-12	1	2,9
13-15	30	85,7
GOS-E		
1-2	7	20,5
3-4	16	47,1
5-6	11	32,3

## Discussion

The prevalence of post-traumatic cerebral venous thrombosis (ptCVT) remains undetermined. Recent evidence indicates that the incidence of ptCVT on venous imaging in patients with a pretest suspicion for sinus injury is 32.3%, with fractures adjacent to the sinus being the primary factor [9,10]. Several studies have demonstrated an unexpectedly high and consistent frequency of traumatic CVT among patients with skull fractures [4,6,10]. Our study unequivocally reveals that venous thrombosis associated with skull fracture is being underdiagnosed. Only 26.2% of our cohort had a fracture near the venous sinuses. However, hyperdensity and hypodensity signal changes in the vicinity of the sinus should raise a high index of suspicion for evolutionary thrombosis. This underscores the fact that not all extra-axial hyperdense hemorrhagic collections are subdural hematomas when detected in the posterior fossa or alongside the interhemispheric fissure, thus warranting careful examination to exclude or confirm concurrent dural sinus injury.

Our study elucidates that CVT is not exclusive to patients with severe head trauma [6]. Notably, 61% of patients initially admitted were classified as mild TBI upon admission. Regarding the preferred location of traumatic venous thrombosis, it predominantly occurs in the lateral venous sinuses, as evidenced by our study (about 95%), aligning with literature findings [11].

In our investigation, the mean time to initiate anticoagulation (AC) was by the fifth-day post-TBI. The optimal timing for screening patients with TBI for CVT remains without solid evidence. It is postulated that the sensitivity of imaging increases over time, even beyond 30 days following injury. Some studies advocate for screening at an interval of five to seven days post-TBI in patients at risk of CVT, as therapeutic AC is unlikely to be initiated earlier in trauma patients. Delayed imaging with CT venogram (CTV) or MR angiogram (MRA) offers the additional advantage of documenting the stability of the contusion prior to therapeutic AC.

It is acknowledged that occlusive thromboses heighten the risk of complications. Nevertheless, our study contributes new insights by demonstrating that non-occlusive venous thromboses are not devoid of complications, with two cases of venous infarction reported in this thrombosis type. This finding contrasts with the study by Delgado et al., which, in a cohort of 44 patients with non-occlusive venous thrombosis, reported no complications [12,13,14].

Our research observed four complications following the commencement of hypocoagulation (three with progression of Intracranial hemorrhage (Figure 3)). The majority of these occurred within two days post-therapy initiation. Consequently, we propose that image repetition in the subsequent 24/48 hours after starting AC may prove beneficial. Remarkably, even the initiation of AC at a prophylactic dose is not devoid of risk for hemorrhagic progression, necessitating cautious consideration [15].

Afshari et al. reported that patients with intraparenchymal hemorrhage (IPH) or acute subdural hematoma (aSDH) should defer AC until stability scans indicate resolution of aSDH or IPH, or progression of aSDH to the subacute or chronic stage, typically withholding AC for ≥7 days post-injury [10].

A limitation of our study is the inability to follow up patients post-discharge, precluding data on the timing and degree of full thrombus recanalization due to the absence of reimaging. Some studies, like that by Kim et al., suggest that young age, partial sinus occlusion, and AC treatment are significant predictors for complete recanalization compared to conservative management [9,16]. There is a prevailing view that asymptomatic patients, showing no thrombus progression, might experience spontaneous recanalization and, therefore, may not require AC treatment.

The ideal duration of treatment is not universally agreed upon; however, a period of three to six months with interval imaging is considered potentially appropriate. A prior study by Hersh et al. reported a 50% resolution rate of thrombosis at three months [12].

This study possesses several limitations, including its single-center, retrospective nature and the small cohort size due to the low incidence and an underestimated prevalence of tCVT.

In summary, patients with TBI exhibiting increased intracranial pressure with lesions proximal to the venous sinuses should be considered for high suspicion and evaluated with specialized venous phase imaging, such as CT or MR venogram, to detect underlying venous sinus thrombosis.

## Conclusions

CVTs, in the context of trauma, are a reality and often appear in lateral venous sinuses. Our suspicion index of concomitant CVT when leading with fractures in the vicinity of the venous sinuses, especially in the lateral sinuses and jugular hole, should be increased.

Mild TBI patients often go unnoticed on the ward. They are not exempt from CVT, and this pathology should be considered in these patients too. Occlusive thrombosis often progresses to complications, and for that, a lower threshold for hypocoagulation should be considered. Our work demonstrated that in non-occlusive venous thrombosis, complications are possible, and, as such, the hypocoagulation in these cases should also be considered. Hypocoagulation, even in the prophylactic dose, is not without complications. A control CT scan in the first 24 to 48 hours after the start of treatment should be done in order to detect complications.

Failure to recognize CVT may explain some unexpected and poor clinical outcomes. Further explanation of the relationship between CVT and outcome in TBI patients will require larger prospective investigations.
